# Knowledge, awareness and use of current practice of palliative care amongst physiotherapists

**DOI:** 10.4102/sajp.v79i1.1786

**Published:** 2023-10-10

**Authors:** Abdulsalam M. Yakasai, Sonill S. Maharaj, Umar M. Gidado, Jibril M. Nuhu, Sani A. Haruna, Musa S. Danazumi

**Affiliations:** 1 Discipline of Physiotherapy, College of Health Sciences, University of KwaZulu-Natal, Durban, South Africa; 2 Department of Physiotherapy, Faculty of Allied Health Sciences, Bayero University, Kano, Nigeria; 3 Department of Biochemistry, Faculty of Basic Medical Sciences, Maitama Sule University, Kano, Nigeria; 4 Department of Physiotherapy, Federal Medical Center, Nguru, Nigeria; 5 Department of Physiotherapy, Faculty of Health Sciences, La Trobe University, Bundoora, Australia

**Keywords:** palliative care physiotherapy, knowledge, awareness, current practice, physiotherapists

## Abstract

**Background:**

Recently, there has been a marked increase in the incidence of cancer, HIV, and other noncommunicable diseases globally. Thus, the demand for palliative care (PC), including end-of-life care, continues to grow worldwide. Physiotherapy has an important role in PC as it aims to alleviate symptoms and improve quality of life by optimising independent levels of function.

**Objective:**

To assess the level of knowledge, awareness and current practice of PC amongst Nigerian physiotherapists in clinical practice.

**Method:**

Our study used a cross-sectional descriptive census-based method and recruited practising physiotherapists in Nigeria. An electronic questionnaire was used to collect data over 12 weeks, comprising 36 semistructured questions in four domains: personal information, knowledge, awareness and current practices regarding PC. Data were analysed using a pragmatist paradigm.

**Results:**

Of the 426 physiotherapists who participated, 50% (*n* = 213) had a postgraduate degree, 49.5% (*n* = 211) had a bachelor’s degree and 0.5% (*n* = 2) had a Doctor of Physical Therapy degree. The results also indicate that 73.9% (*n* = 315) of the participants had sufficient knowledge about PC, 80.5% (*n* = 343) had a sufficient level of awareness about PC and 66.7% (*n* = 284) were using current clinical practices in the rehabilitation of critically ill patients or those with chronic conditions.

**Conclusion:**

It was concluded that most Nigerian physiotherapists had sufficient knowledge and awareness about PC and were involved in the management of patients requiring PC physiotherapy.

**Clinical implication:**

It can be understood that a large proportion of Nigerian physiotherapists have clinical experience managing patients requiring PC, despite inadequate formal training in this field.

## Introduction

Recently, there has been increasing concern and need for more effective care for people with complex diseases and those with terminal illnesses. Palliative care is a specialised medical care aimed at providing pain relief and management of distressing and debilitating symptoms, optimising quality of life and mitigating suffering associated with life-threatening illnesses faced by patients and their families (NHS Choices 2017). This is achieved through early assessment, diagnosis and intervention to manage pain and other symptoms, as well as incorporating social, psychological and spiritual aspects of holistic care (Prevost & Grach 2012). According to Sujatha and Jayagowri (2017), palliative care is not restricted to cancer or terminally ill patients, as it was previously. The focus has now been broadened to include caring for patients with diseases that last for many years and those with various life-threatening conditions. In addition, palliative care now covers from the point of diagnosis to disease progression and end-of-life care (Murray et al. 2015). Palliative care also offers a support system for carers to cope during the patient’s illness and in bereavement after the loss of a relative or friend (Addington-Hall & Higginson 2011).

Advances in medical care have improved the survival of people around the globe and have resulted in an increase in the number of people reaching old age (Payne, Coyne & Smith 2002). Nigeria is amongst the low- to middle-income countries that are worst hit by deaths from noncommunicable diseases, according to the first World Health Organization (WHO) Global Status Report on noncommunicable diseases. The diseases with a rising burden in Nigeria include cancer, coronary artery diseases, chronic metabolic diseases, sickle cell disease, chronic respiratory diseases, stroke and mental disorders (Aregbeshola 2012). Individuals presenting with these chronic conditions or their comorbidities alongside those with terminal illnesses could benefit from palliative care physiotherapy. Palliative care has a multidisciplinary approach and is provided by specially trained health professionals, including medical practitioners, nurses, physiotherapists, pharmacists and many others within the healthcare system (Sujatha & Jayagowri 2017).

Physiotherapy is concerned with optimising movement and achieving maximal possible function through preventive and curative means as well as rehabilitation (Kumar & Jim 2010). Despite the potential benefits of palliative care physiotherapy, evidence suggests a lack of palliative care for patients suffering from chronic conditions and end-of-life illnesses receiving physiotherapy treatment (Chartered Society of Physiotherapy 2004). As professionals, physiotherapists should have adequate knowledge, be well trained and have the skills to render palliative care physiotherapy for terminally ill patients and those living with chronic conditions from diagnosis to disease progression and the end-of-life stage (Chiarelli, Johnston & Osmotherly 2014). In Nigeria, awareness of palliative care is low even decades after its introduction (Daniel Chukwunyere 2019). Moreover, data concerning knowledge of Nigerian physiotherapists about palliative care are rarely documented, and it is not known whether they use palliative care physiotherapy in the rehabilitation of terminally ill patients and those living with chronic conditions. Our study examined the existing knowledge, awareness and practices of palliative care by registered physiotherapists working in Nigeria.

## Method

This was a cross-sectional survey using an online questionnaire, conducted to explore the knowledge, awareness and current practices of physiotherapists in palliative care physiotherapy. Our study was conducted amongst Nigerian physiotherapists working in various governmental and nongovernmental hospitals and clinics. The anonymised participants were recruited from the database of the Medical Rehabilitation Therapists (Registration) Board of Nigeria (MRTB). According to the inclusion criteria, participants (1) were either male or female, (2) had a minimum of bachelor’s degree in physiotherapy, (3) were currently residing and practising physiotherapy in Nigeria. Physiotherapists who were not registered with the MRTB were not included. A census-based method was used for our study, as the census is a method that gathers information about every member of the population. It provides intensive and in-depth information covering many facets of the problem in question (Lavrakas 2013).

### Questionnaire design and validation

Our survey was based on an electronic structured questionnaire using Google Forms, developed by expert physiotherapists in the field of palliative care. The questionnaire consisted of 36 questions and four parts, namely (1) personal information (comprising 7 questions); (2) knowledge about palliative care physiotherapy (comprising 11 questions); (3) awareness about palliative care physiotherapy (comprising 6 questions); and (4) practices of palliative care physiotherapy (comprising 12 questions). Ten physiotherapy experts in the field of palliative care were piloted to check the content and face validity and appropriateness for the local context of the questionnaire. Then a cognitive debriefing session was held and recommendations from the participants were received for improving the quality of the survey. Appropriate amendments were made by members of our research team to ensure that the content was in line with the intended meaning and well suited to the aim and context of the study (Terwee et al. 2007). In addition, the questionnaire was equally assessed for divergent validity, as 20 physiotherapists completed it together with another questionnaire, the Physical Therapy in Palliative Care – Knowledge, Attitudes, Beliefs and Experiences (PTiPC-KABE) Scale (Morrow et al. 2017). Pearson’s Product Moment Correlation (PPMC) indicated an insignificant relationship between the two questionnaires [0.013 (*n* = 20, *p* = 0.893)], showing that the two questionnaires measured entirely different constructs. In order to establish test–retest reliability, 15 physiotherapists filled in the questionnaire at a 2-week interval. The result indicated a highly significant intra-class correlation coefficient [ICC = 0.788 (*n* = 20, *p* = 0.001)], indicating that the questionnaire is reproducible. The link to the final version of the online questionnaire was distributed to the participants via e-mail and WhatsApp. Reminder e-mails were sent every week to the participants in order to have a high response rate.

### Statistical analysis

A pragmatist paradigm was used to analyse data for both qualitative-constructivist and quantitative-positivistic approaches. The mixed methods data analysis used in our study was to give a broader perspective and more in-depth analysis of the subject matter (Kaushik & Walsh 2019).

### Qualitative analysis

Responses were scored as described in the study by Yakasai et al. (2020) using the scoring system as follows: agree, disagree and undecided. The questionnaire texts were analysed qualitatively using content analysis (Shelley & Krippendorff 2012). Firstly, the authors read the questions several times to have a better understanding of the text and to maintain the meaning of the content according to the local context. Secondly, two of the authors performed coding separately by combining the questions into units which were labeled as codes (i.e., group of words with similar meanings and/or connotations). The codings were compared, discussed and consensus was reached amongst the authors. Thirdly, the codes were synthesised and grouped into meaningful subcategories and labeled with appropriate titles: knowledge, awareness and practice. Finally, the codes were subcategorised and merged into broader categories and then pooled into major themes: sufficient knowledge, insufficient knowledge, lack of knowledge; sufficient awareness, insufficient awareness, lack of awareness; and standard practice, substandard practice and lack of practice.

### Quantitative analysis

The Shapiro–Wilk test was used to assess the normality of the data, whilst Levene’s test was used to assess the homogeneity of variance. As the data were normally distributed, PPMC was used to examine the relationship between knowledge, awareness and practice of palliative care, whilst a one-way analysis of variance (ANOVA) was used to determine the differences between the outcomes as dependent variables (knowledge, awareness and palliative care practice) and independent variables (novice, experienced and expert professionals). Bonferroni post-hoc analysis was conducted to determine where a significant difference exists. An alpha level was set at 0.05 and confidence interval at 95%. All data were analysed using SPSS 24.0 (SPSS, Inc.).

### Ethical considerations

Our study was approved by the institutional review board where the study was conducted (reference number: NHREC/06/12/19/79). The participants were informed that clicking the link and assessing the questionnaire was taken as providing consent to participate in our study, as the informed consent form was attached to the questionnaire.

## Results

### Demographic characteristics of the physiotherapists

Participants were divided into three predefined age groups that covered 191 (44.8%) young adults, 164 (38.5%) middle-aged adults and 71 (16.7%) older adults as described by Petry (2002) (see Table 1). In total, 426 physiotherapists participated in our study; 68.8% (*n* = 293) were practising in government hospitals with 31.2% (*n* = 133) practising in private hospitals and nongovernmental organisations. With regard to educational qualification, 50% (*n* = 213) of the physiotherapists had postgraduate degrees, 49.5% (*n* = 211) had a bachelor’s degree in physiotherapy and only 0.47% (*n* = 2) had a Doctor of Physical Therapy degree. In addition, 189 (44.4%) participants were within the first decade of their professional career (novice professional period), 150 (35.2%) were within the second decade of their professional career (experienced period) and 87 (20.4%) were within the third decade of their professional career (expertise).

**TABLE 1:** Demographic characteristics of the participants (*n* = 426).

Demographics	*n*	%	Mean	s.d.
**Q1: Age:**	-	-	37.83	± 6.95
Young adults (19–35 years)	191	44.9	-	-
Middle-aged adults (36–55 years)	164	38.4	-	-
Older adults (above 55 years)	71	16.7	-	-
**Q2: Gender:**			-	-
Male	258	60.6	-	-
Female	168	39.4	-	-
**Q3: Work setting:**				
Government hospital	293	68.8	-	-
Private hospital	133	31.2	-	-
**Q4: Education level:**				
Doctor of Philosophy	57	13.4	-	-
Master of Science	156	36.6	-	-
Doctor of Physiotherapy	2	0.5	-	-
Bachelor of Physiotherapy	211	49.5	-	-
**Q5: Practice level:**				
1^st^ decade of practice	189	44.4	-	-
2^nd^ decade of practice	150	35.2	-	-
3^rd^ decade of practice	87	20.4	-	-
**Q6: Rank:**				
Physiotherapist	185	43.4	-	-
Senior physiotherapist	101	23.7	-	-
Principal physiotherapist	96	22.5	-	-
Chief physiotherapist	32	7.5	-	-
Assistant director of physiotherapy services	10	2.4	-	-
Deputy director of physiotherapy services	2	0.5	-	-
**Q7: Speciality:**				
Orthopaedic physiotherapist	157	36.9	-	-
Neurological physiotherapist	99	23.2	-	-
Cardiopulmonary physiotherapist	53	12.4	-	-
Geriatric physiotherapist	41	9.6	-	-
Paediatric physiotherapist	66	15.5	-	-
Women’s health physiotherapist	10	2.47	-	-

s.d, standard deviation.

### Findings on physiotherapists’ knowledge about palliative care

Findings on the knowledge of physiotherapists about palliative care were assesssed and presented as number of codes and themes that emerged as follows: sufficient knowledge, insufficient knowledge and lack of knowledge (Table 2). After summation of the codes, 73.9% (*n* = 315) of the physiotherapists had ‘sufficient knowledge’, 20.7% (*n* = 88) of them had ‘insufficient knowledge’ and 5.4% (*n* = 23) showed ‘lack of knowledge’. Physiotherapists in the sufficient knowledge group had adequate knowledge about palliative care physiotherapy. Those in the insufficient knowledge group had some knowledge but inadequate awareness. Physiotherapists in the lack of knowledge group had no knowledge about palliative care physiotherapy.

**TABLE 2:** Physiotherapists’ responses on knowledge, awareness and current practice of palliative care physiotherapy (*n* = 426).

Question	Response	*n*	*%*
**Knowledge questions**			
Q1: Palliative care is an interdisciplinary medical care approach aimed at optimising the quality of life and mitigating suffering amongst people with serious and complex illnesses.	Agree	346	81.2%
	Disagree	80	18.8%
	Undecided	0	0%
Q2: Palliative care provides relief from symptoms and stress for patients with life-threatening and life-limiting illnesses and their families.	Agree	299	70.2%
	Disagree	123	28.9%
	Undecided	4	0.9%
Q3: Palliative care goals are through prevention, early identification and relief of physical (including pain), psychological, psychosocial and spiritual suffering and by optimising independent function to improve health-related quality of life.	Agree	326	76.5%
	Disagree	6	1.4%
	Undecided	94	22.1%
Q4: Palliative care was initially developed in response to the needs of cancer or terminally ill persons alone.	Agree	300	70.4%
	Disagree	126	29.6%
	Undecided	0	0%
Q5: Palliative care is now applicable to diseases that last for many years and other life-threatening conditions that remain symptomatic and have functional limitations.	Agree	89	20.9%
	Disagree	312	73.2%
	Undecided	25	5.7%
Q6: Palliative care is appropriate at any age and any stage of a serious illness and can be provided together with curative treatment.	Agree	193	45.3%
	Disagree	201	47.2%
	Undecided	32	7.5%
Q7: A number of chronic life-threatening conditions are prevalent in Nigeria, including HIV, cerebrovascular disease, diabetes mellitus and chronic pulmonary disease. People presenting with these conditions or their sequelae could benefit from a palliative care approach.	Agree	423	99.3%
	Disagree	0	0%
	Undecided	3	0.7%
Q8: Palliative care begins at any time of a serious disease, most especially at the time of the diagnosis process, and continues through cure or until death and then into bereavement.	Agree	201	47.2%
	Disagree	151	35.4%
	Undecided	74	17.4%
Q9: Palliative care is not against the values of physiotherapy.	Agree	214	50.3%
	Disagree	212	49.8%
	Undecided	0	0%
Q10: Physiotherapists have an important role in the palliative care team, providing symptom management and improving quality of life by optimising independent levels of function.	Agree	370	86.9%
	Disagree	0	0%
	Undecided	56	13.1%
Q11: Palliative care is provided by a primary care provider and is supported by a team of specialists, usually a physician, nurse, physiotherapist, social worker and spiritual care counselor.	Agree	109	25.6%
	Disagree	0	0%
	Undecided	317	74.4%
**Awareness questions**			
Q1: Palliative care is as important as curative care in physiotherapy practice.	Agree	301	70.6%
	Disagree	77	18.1%
	Undecided	48	11.3%
Q2: Physiotherapists are aware of palliative care to patients who are terminally ill and their families.	Agree	345	80.9%
	Disagree	81	19.1%
	Undecided	0	0%
Q3: There is societal support for and awareness of the need for physiotherapy in palliative care.	Agree	256	60.1%
	Disagree	70	16.4%
	Undecided	100	23.5%
Q4: Physiotherapists are well qualified to support and communicate with terminally ill or dying patients and their families.	Agree	404	94.8%
	Disagree	0	0%
	Undecided	22	5.2%
Q5: Palliative care is necessary for physiotherapy education in Nigeria.	Agree	426	100%
	Disagree	0	0%
	Undecided	0	0%
Q6: Curative care is more important than palliative care in the physiotherapy environment.	Agree	0	0%
	Disagree	208	48.8%
	Undecided	218	51.2%
**Current practice questions**			
Q1: Do medical doctors refer patients for physiotherapy palliative care?	Yes	169	39.7%
	No	51	11.9%
	Maybe	206	48.4%
Q2: Do medical staff support physiotherapists in palliative care in your workplace?	Agree	70	16.4%
	Disagree	0	0%
	Undecided	356	83.7%
Q3: Do you refer or invite other health care professionals if the need arises in the course of managing patients who require physiotherapy palliative care?	Agree	426	100%
	Disagree	0	0%
	Undecided	0	0%
Q4: List any three conditions that require palliative care in your specialty.	Complete response	121	28.4%
	Incomplete response	67	15.7%
	No response	238	55.9%
Q5: If you refer patients who will benefit from palliative care to other health professionals, to whom do you refer?	Oncologist	207	48.6%
	Neurologist	3	0.7%
	Nephrologists	111	26.1%
	Cardiologists	7	1.7%
	Pulmonologist	98	23.0%
Q6: TENS, heat, massage, lymphedema treatment and acupuncture are common forms of pain relief modalities and are employed often in palliative care.	Agree	189	44.4%
	Disagree	0	0%
	Undecided	237	55.6%
Q7: Therapeutic massage, cold therapy, TENs, range of motion, strengthening exercises and gait training are employed for treating patients with cancer.	Agree	226	53.1%
	Disagree	0	0%
	Undecided	200	46.9%
Q8: Psychological and physical activity-based interventions have been proven to be better in improving the quality of life in patients with cancer-related fatigue in palliative care.	Agree	367	86.2%
	Disagree	0	0%
	Undecided	59	13.8%
Q9: Physical activity is indirectly associated with improved quality of life through pathways that include fatigue, pain, social support and self-efficacy in individuals with multiple sclerosis in palliative care.	Agree	226	53.1%
	Disagree	0	0%
	Undecided	200	46.9%
Q10: Improvements in social support, self-efficacy and reduced functional limitations following physical activity programme in multiple sclerosis patients are important. Besides drugs, physiotherapy is the mainstay in the management of spasticity in multiple sclerosis in palliative care.	Agree	301	70.7%
	Disagree	0	0%
	Undecided	125	29.3%
Q11: Aerobic and resistance exercise training programmes decrease pain, depression and stress, and increase the quality of life and better physical self-concept in patients with spinal cord injury in palliative care.	Agree	174	40.9%
	Disagree	201	47.2%
	Undecided	51	11.9%
Q12: Breathing retraining, such as diaphragmatic breathing or pursed lip breathing, are useful in palliative management of dyspnoea.	Agree	221	51.9%
	Disagree	0	0%
	Undecide	205	48.1%
Q13: Positioning techniques, relaxation, breathing awareness exercises, walking and stair climbing activities, coping and pacing, and activity modification are useful in physiotherapy management of breathlessness in palliative care.	Agree	226	53.1%
	Disagree	0	0%
	Undecided	200	46.9%

### Findings on physiotherapists’ awareness about palliative care

The number of codes for awareness about palliative care physiotherapy and the themes that emerged are illustrated in Table 3. The codes were labelled and categorised into three major themes as follows: adequate awareness, inadequate awareness and lack of awareness. After summation of the codes, 80.5% (*n* = 343) of the physiotherapists had ‘adequate awareness’, 13.4% (*n* = 57) had ‘inadequate awareness’ and 6.1% (*n* = 26) showed ‘lack of awareness’. Adequate awareness indicated that the participants were well aware of palliative care physiotherapy. Inadequate awareness indicated that the participants had some level of awareness, although inadequate, and lack of awareness indicated that the participants were not aware of palliative care physiotherapy.

**TABLE 3:** Themes and number of codes for physiotherapists’ knowledge, awareness and current practice (*n* = 426).

Themes	Number of codes	*n*	%
**Physiotherapists’ knowledge (*n* = 426)**			
Sufficient knowledge	8610	315	73.9
Insufficient knowledge	2422	88	20.7
Lack of knowledge	605	23	5.4
**Physiotherapists’ awareness (*n* = 426)**			
Sufficient knowledge	5196	343	80.5
Insufficient knowledge	872	57	13.4
Lack of knowledge	388	26	6.1
**Physiotherapists’ practice (*n* = 426)**			
Standard practice	8148	284	66.7
Substandard practice	520	18	4.2
Lack of practice	3567	124	29.1

Q, question.

### Findings on physiotherapists’ practice of palliative care

The number of codes for practice of palliative care physiotherapy and the themes that emerged are illustrated in Table 3. The codes were labelled and categorised into three major themes as follows: standard practice, substandard practice and lack of practice. After summation of the codes, 66.7% (*n* = 284) of the physiotherapists used ‘standard practice’, 4.2% (*n* = 18) used ‘substandard practice’ and 29.1% (*n* = 124) had ‘lack of practice’. Standard practice indicated that the participants had adequate skills in the management of palliative care patients. Substandard practice indicated that participants in this group had inadequate skills in the management of palliative care patients, and lack of practice indicated that the participants in this group had no skills in managing patients in need of palliative care.

### Correlation between physiotherapists’ knowledge, awareness and practice of palliative care

The relationship between physiotherapists’ knowledge, awareness and practice of palliative care is presented in Table 4. The results indicate that knowledge was moderately and strongly related with awareness (*r* = 0.68, *p* = 0.001, *n* = 426) and practice of palliative care (*r* = 0.85, *p* = 0.001), respectively. Additionally, practice of palliative care was strongly related with awareness (*r* = 0.74, *p* = 0.001, *n* = 426).

**TABLE 4:** Spearman’s rank correlation between therapists’ knowledge, awareness and practice (*n* = 426).

Variables	Knowledge		Awareness		Practice	
	*r*	*p*	*r*	*p*	*r*	*p*
Knowledge	-	-	0.68	0.001	0.85	0.001
Practice	-	-	0.74	0.001	-	-

*r*, coefficient of correlation; *p*, level of significance.

### Findings on differences in knowledge, awareness and practice of palliative care

The one-way ANOVA was carried out to determine differences in knowledge, awareness and practice of palliative care (Table 5). The results indicated statistically significant differences in knowledge (*F* = 14.43, *df* = 2.423, *p* = 0.0001), awareness (*F* = 6.32, *df* = 2.423, *p* = 0.0001) and practice of palliative care (*F* = 10.21, *df* = 2.423, *p* = 0.0001), with a significant linear trend for knowledge (*p* = 0.0001), awareness (*p* = 0.014) and practice of palliative care (*p* = 0.003) to increase with higher professional experience. Post-hoc analysis indicated statistically significant differences with large effect sizes in the independent variables only between novice and expert, with experts having more knowledge [mean difference = 7.22 (3.35, 10.12), effect size (ɳ^2^) = 0.768, *p* = 0.023], awareness [mean difference = 3.48 (2.35, 6.82), effect size (ɳ^2^) = 0.711, *p* = 0.007] and palliative care practice [mean difference = 7.01 (3.66, 9.87), effect size (ɳ^2^) = 0.862, *p* = 0.003] than novice physiotherapists. The effect sizes (ɳ^2^) indicate 76.8%, 71.1% and 86.2% of the variation in knowledge, awareness and the practice of palliative care by expert professionals.

**TABLE 5:** One-way ANOVA for the difference in knowledge, awareness and current practice (*n* = 426).

Professional experience	*n*	Mean	s.d.	*F*	*df*	*p*-value	*p*-value trend
**Difference in knowledge (*n* = 426)**	-	-	-	14.43	2.423	0.0001	0.0001
Novice	189	9.67	2.12	-	-	-	-
Experienced	150	10.92	2.87	-	-	-	-
Expert	87	16.89	1.95	-	-	-	-
**Difference in awareness (*n* = 426)**	-	-	-	6.32	2.423	0.002	0.014
Novice	189	3.88	0.06	-	-	-	-
Experienced	150	5.03	0.21	-	-	-	-
Expert	87	7.34	0.16	-	-	-	-
**Difference in current practices (*n* = 426)**	-	-	-	10.21	2.423	0.0001	0.003
Novice	189	4.87	1.77	-	-	-	-
Experienced	150	8.01	2.63	-	-	-	-
Expert	87	11.88	2.37	-	-	-	-

*N*, number of physiotherapists; s.d., standard deviation; *F*, ANOVA; *df*, degree of freedom; ANOVA, analysis of variance.

## Discussion

Palliative care is often compared to and considered synonymous with pain medicine, geriatric medicine and rehabilitative medicine. However, this form of care has evolved to include rehabilitation of individuals with serious illnesses, having team members including medical practitioners, nurses, pharmacists, physiotherapists and other health professionals having assigned roles. Although physiotherapists may not be formally trained to become specialists in palliative care in low- and middle-income countries, there are special interest groups whose members focus on palliative care and have garnered experience over the years in the management of patients requiring palliative care in these countries. Despite this, there is a lack of evidence concerning knowledge, awareness and practice of palliative care amongst physiotherapists in sub-Saharan Africa, particularly Nigeria. To the best of the authors’ knowledge, there are no studies available on the knowledge, awareness and practice of palliative care amongst Nigerian physiotherapists.

The results of our study show that more than half of the surveyed physiotherapists (73.9%) had sufficient knowledge about palliative care physiotherapy. This could be because of the increase in the number of patients who require palliative care services in the nation, such as patients with cancer and stroke across Nigeria (Budkaew & Chumworathayi 2013). Our findings also indicate that some physiotherapists had insufficient knowledge and a few lacked knowledge of palliative care physiotherapy. The current findings are somewhat in agreement with those of Morrow et al. (2017), who reported amongst physiotherapists in South Africa. It was suggested that the knowledge of the scope of palliative care is still limited, and this suggestion is supported by the moderate score (56%) of the ‘palliative care knowledge’ domain. These contradict the results of a recent study by De Oliveira, Rodrigues and Barreto (2021) conducted on the knowledge of Brazilian physiotherapists on palliative care in paediatrics. The results indicated that only 34.1% of physiotherapists reported having adequate knowledge about palliative care physiotherapy.

The participants in our study indicated an adequate level of awareness (80.5%) about palliative care physiotherapy. A high level of knowledge about palliative care is considered an important factor in the development of positive awareness toward palliative care. However, the reason for this adequate level of awareness may be related to continuing professional development programmes organised by the palliative care physiotherapy specialty group in Nigeria. Our findings are similar to the results of previous studies. Mawani et al. (2015) assessed the awareness of palliative care amongst physiotherapy students. About 80% of the participants strongly agreed that palliative care is as important as curative care in physiotherapy, and 70% agreed that palliative care should be included in the course content of physiotherapy education. Akinyemiju et al. (2015) surveyed palliative care awareness amongst health professionals. Their results indicate that 98% of the professionals had adequate awareness about palliative care. Sujatha and Jayagowri (2017) assessed palliative care awareness amongst undergraduate health care students. They concluded that 70% of students from the medical college were aware of palliative care.

Our study revealed that 66.7% of the participants adopted the practice of palliative care physiotherapy. This indicates that the majority of the physiotherapists had adequate skills and/or training in the rehabilitation of terminally ill patients. This result supports the findings by Morrow et al. (2017) on the practice of palliative care amongst physiotherapists in South Africa. The results show that physiotherapists apply standard current practices in the management of patients needing palliative care speciality.

Our results also indicate that knowledge was moderately and strongly correlated with awareness and application of palliative care practices. The findings also indicate significant differences in knowledge, awareness and practices of palliative care physiotherapy between the expert and the novice, with experts having more knowledge and awareness and practising palliative care more in the rehabilitation of individuals with chronic illness or the terminally ill. In addition, our finding that significant linear trends for knowledge, awareness and practices of palliative care increase with increasing professional experience is similar to the work of Morrow et al. (2017). Their results indicated a significant association with years of experience on the ‘attitudes of physiotherapy in the palliative care’ domain, with a negative correlation between number of years qualified and percentage score (Spearman *r* = −0.14; *p* = 0.03). However, a study by Yakasai et al. (2020) indicated that expert physiotherapists have better knowledge and better practise compared to novice physiotherapists. Similar findings were also reported in previous studies, which indicated that expert physiotherapists have better knowledge and also use better reflection methods in the clinical decision-making process when compared to novice physiotherapists (Case, Harrison & Roskell 2000; Doody & McAteer 2002; May et al. 2013; Wainwright et al. 2010).

### Limitations of our study

Our study is not without limitations. Firstly, it was conducted in Nigeria and may not be generalisable to other settings. Secondly, the design was cross-sectional and cannot therefore establish cause and effect.

### Implications for physiotherapists

Our findings suggest that physiotherapists have adequate knowledge and awareness and use palliative care physiotherapy. However, mentorship and workshops on palliative care physiotherapy are needed for novice clinicians. Thus, palliative care physiotherapy should be included in undergraduate curricula.

## Conclusion

Based on the findings, it can be concluded that a large proportion of Nigerian physiotherapists have knowledge, awareness and clinical experience in managing patients requiring palliative care physiotherapy. Furthermore, expert physiotherapists have better knowledge and awareness and also use current practices more in palliative care physiotherapy compared to novice physiotherapists.
